# Visualization techniques of the inferior alveolar nerve (IAN): a narrative review

**DOI:** 10.1007/s00276-015-1510-z

**Published:** 2015-07-12

**Authors:** Annelies Weckx, Jimoh Olubanwo Agbaje, Yi Sun, Reinhilde Jacobs, Constantinus Politis

**Affiliations:** Department of Oral and Maxillofacial Surgery, Faculty of Medicine, Catholic University Leuven, University Hospitals Leuven, Kapucijnenvoer 33, 3000 Leuven, Belgium; OMFS-IMPATH Research Group, Department of Imaging and Pathology, Faculty of Medicine, Catholic University Leuven, Kapucijnenvoer 33, 3000 Leuven, Belgium

**Keywords:** Inferior alveolar nerve, Visualization, Magnetic resonance imaging, Cone-beam computed tomography, Panoramic radiography, Endoscopy

## Abstract

**Purpose:**

The purpose of this study was to produce an overview of the present visualization techniques of the inferior alveolar nerve (IAN) in order to reduce the rates of nerve damage after third molar (M3) removal and bilateral sagittal split osteotomy (BSSO).

**Methods:**

An electronic literature search was performed of the English-language scientific literature published prior to December 31, 2014 using the LIMO KU Leuven search platform. Information on the specifications of the different imaging techniques, their clinical application, advantages, disadvantages, and duration was extracted from 11 reports.

**Results:**

Five methods for IAN visualization were obtained from the search results, which are cone-beam computed tomography (CBCT) and automatic extraction of the IAN canal using computed tomography (CT), magnetic resonance imaging (MRI), panoramic radiography, endoscopy, and ultrasonographic visualization.

**Conclusion:**

The results of this study suggest that high-resolution MRI is the most commonly used method for direct visualization of the IAN. Six out of the eleven manuscripts use this technique. Recently, there have been some (experimental) modifications to the conventional MRI in the form of diffusion tensor tractography (DTT), phase-contrast magnetic resonance angiography (PC-MRA), and dental MRI. Future studies will focus on an intraoperative application of MRI to visualize the IAN during surgery.

## Introduction

Both removal of third molars (M3) and bilateral sagittal split osteotomy (BSSO) are common procedures in orthognathic surgery [1, 33, 44]. M3 removal and BSSO are performed in a close proximity to the inferior alveolar nerve (IAN) and can therefore cause damage to the IAN. The incidence of temporarily impaired IAN function after M3 removal ranges from 1 to 7 % [22, 41]. Permanent injury is much less frequent at 0.01–2 % [33]. Libersa et al concluded from a 10-year follow-up of claims to a practitioner insurance company that the removal of impacted M3s is the main cause of IAN damage (40.8 %), followed by endodontic treatment (35.3 %), surgical removal of other teeth/cysts (20.7 %), and implant placement (3.2 %). Moreover, M3 removal and implant placement are the main causes for permanent nerve injury [19]. After BSSO, 1.3–18 % of all patients suffer from IAN damage [1]. This causes numbness and loss of sensation in the lower lip, the chin, and the lower teeth. Moreover, many people with permanent IAN injury suffer from neuropathic pain [29]. As the latter cannot be fully resolved, the patient is most often left with lifelong inconvenience and pain [33].

The rates of IAN preservation and postoperative nerve damage could improve with better pre- and perioperative identification of the IAN. This is of considerable importance, as most patients needing elective surgery in the vicinity of the IAN are young and in perfect health. At the same time, maxillofacial surgeons express a need for adequate visualization of the IAN during surgery to avoid nerve damage. Thus, an adequate visualization of the IAN pre- and perioperatively could yield a more predictable treatment outcome with less postoperative complications. To obtain an overview of the existing visualization techniques, we reviewed the available literature. The current review is narrative in nature as neither the methodological reports nor the case series on neuropathic problems allow a strict systematic reviewing procedure. The narrative review will therefore rather describe the different visualization methods, their advantages and disadvantages, as well as their clinical applicability in the pre- and perioperative nerve injury risk assessment.

## Materials and methods

### Search strategy

An online search was performed in the LIMO database, a database of the Catholic University of Leuven, which includes publisher databases, Pub Med, IEEE, etc. The search covered all articles published prior to 31st of December 2014.

The following key words were searched: (“inferior alveolar nerve” OR “mandibular nerve”) AND (“visualization” OR “imaging” OR “magnetic resonance imaging” OR “MRI” OR “fluorescence imaging” OR “computed tomography” OR “CT” OR “CBCT” OR “cone beam computed tomography” OR “cone beam CT” OR “3-dimensional” OR “3D”).

The results of a manual search of the reference list of the included publications were added to the electronic search. Reports in the grey literature (information not appearing in the periodic scientific literature obtained from a library, the Internet, or by ordering) were not pursued.

### Selection of studies

The criteria for inclusion of reports for further processing are presented in Table 1. Duplicate reporting was an exclusion criterion.

**Table 1:** Inclusion criteria

Articles written in English language
Human and animal studies
Original study: randomized or non-randomized controlled trial, cohort studies, case control studies, case reports
Availability of full text for assessment
All articles had to be related to visualization of the inferior alveolar nerve (IAN visualization)

One investigator performed the literature search including the selection of titles, abstracts, and full-text publications. All publications obtained by the search were screened by two investigators for meeting the selection criteria. The full text was only reviewed in case the title and the abstract did not contain adequate information for inclusion or exclusion. The amount of evidence and relevance of the findings served as a basis for the final selection of the publications. Any disagreement regarding inclusion was resolved by discussion between the two investigators.

### Data extraction

The full texts of the selected papers were reviewed and the following information was extracted: specifications of the IAN visualization method, clinical application, advantages, drawbacks, and duration.

The extracted data were synthesised by the lead author and the full text was discussed with all co-authors.

## Results

**Fig. 1:**
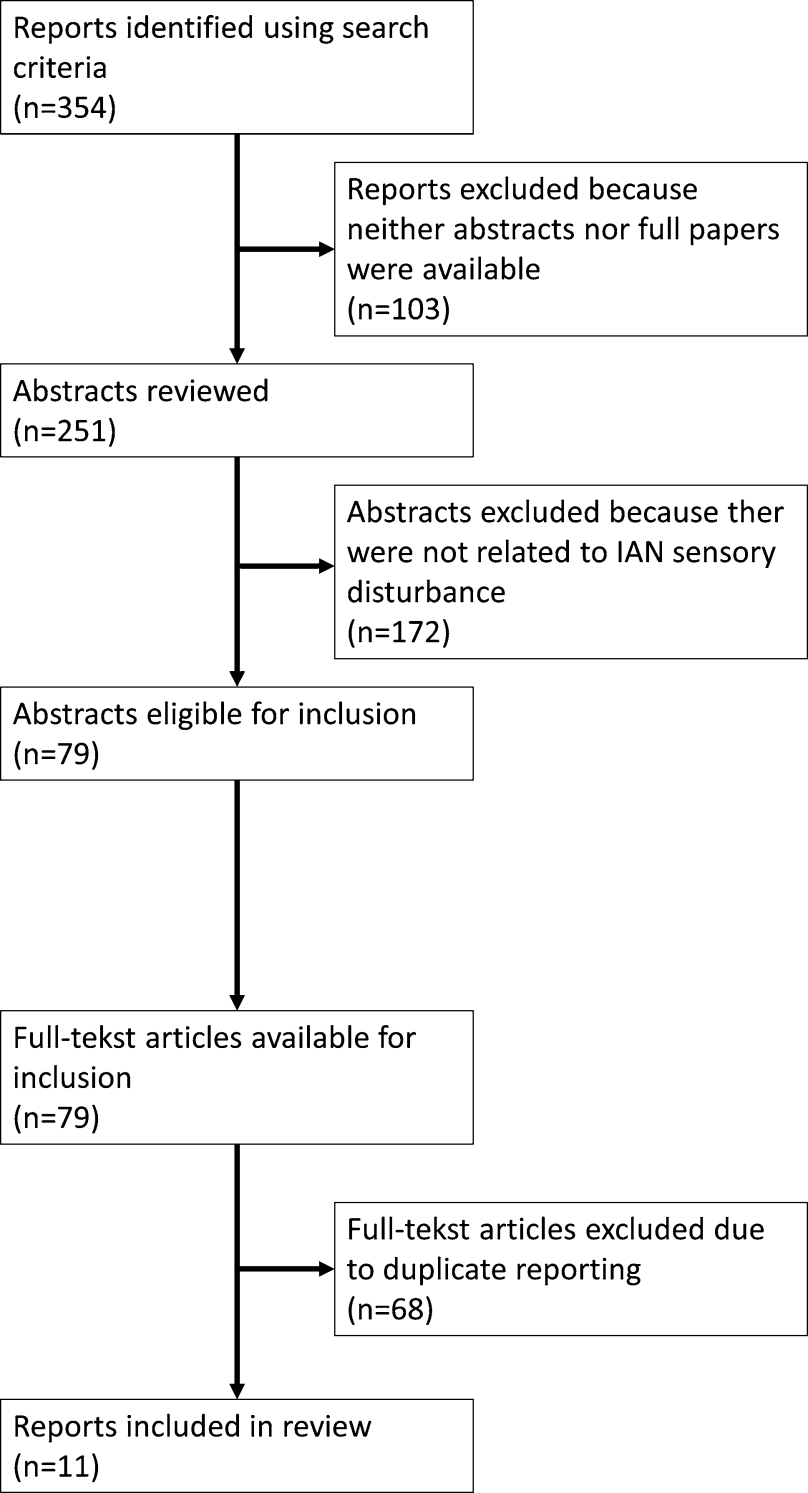
Flow chart of article selection process

Using the aforementioned search strategy, a total of 354 reports were identified. Of this, 103 reports were eliminated because neither abstracts nor full papers were available. Of the remaining 251 abstracts, 172 were excluded because they were not related to IAN visualization. In this way we retrieved 79 full-text articles for scoring. Due to duplicate reporting, another 68 articles were eliminated. Finally, a total of 11 reports were included in this review. The flow chart of the article selection is presented in Fig. 1.

From the search results, five methods for IAN visualization were obtained which are Magnetic Resonance Imaging (MRI) [2, 9, 11, 23, 26, 36], cone-beam computed tomography (CBCT), and automatic extraction of the IAN canal using computed tomography (CT) [9, 16, 17, 43], panoramic radiography [16], endoscopy [3], and ultrasonographic visualization [8].

In 2 articles, more than one visualization method was used: CBCT in combination with MRI or panoramic radiography, respectively [9, 15].

## Discussion

**Fig. 2:**
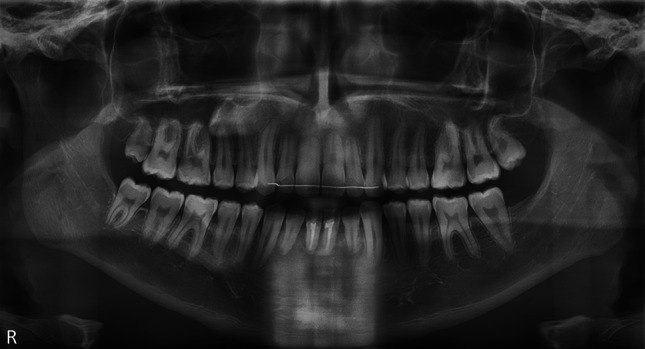
Panoramic radiography of a mandible with insufficient IANC corticalization

In the oral and maxillofacial practice, imaging of the IAN is mostly performed by evaluation of the IAN canal (IANC) using panoramic radiography and (CB) CT images [21, 22, 41]. The IANC appears radiolucent on radiograph, usually with well-defined corticated borders, and is located inferior to the roots of the mandibular teeth [30]. However, these radiologic examinations only evaluate the morphological and bony wall characteristics of the canal, while being unable to directly visualize the IAN itself [11, 24]. The latter becomes a true visualization problem in cases where a clear delineation of the IANC is not possible because of lacking canal corticalization (Fig. 2) [9, 10]. De Oliveira-Santos et al observed that 41 % of the canals are not corticalized. In 23 % this group, the canal on CBCT could be visualized despite the absence of corticalization, but the remainder (18 %) could not be detected [10]. Variations in the location and position of the IANC, including the presence of a bifid mandibular canal, double mental foramen, and the presence of an anterior loop or incisive branch of the mandibular canal, have been well explained in the literature [27, 28, 30, 31]. Moreover, Shiratori et al described that the absence of cortication is more commonly associated with IAN injury after M3 removal [37]. Furthermore, there are cases in which visualization of the nerve rather than the canal is necessary. The latter may be important to evaluate perineural tumor spread or in pathological states of the nerve, but also to prepare for mandibular surgery (e.g., M3 removal or BSSO) in order to minimize the risk of IAN injury [11, 21, 23, 26]. It is important to identify the locations and any anatomical variations of the IANC prior to mandibular surgery. To visualize the nerve itself, high-resolution MRI is the recommended imaging modality [2, 6, 9, 11, 24, 26, 29].

Based on the results above, MRI is the most common method for visualizing the IAN. Six out of the eleven manuscripts use this technique [2, 9, 11, 23, 26, 36].

MRI and the other four imaging modalities described above, with their technical specifications, clinical applications, advantages, and drawbacks, are discussed below.

## MRI

**Fig. 3:**
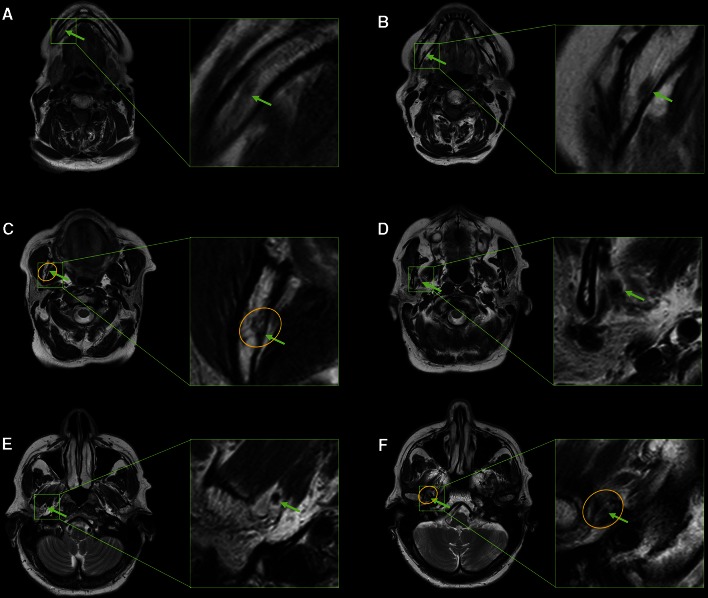
Serial axial 3T-MRI slices (T2w turbo spin-echo sequence) showing the course of the healthy IAN (*green arrows*) from the mandible (**a**) through the mandibular foramen (**c**, *yellow* *circle*) to the foramen ovale (**f**, *yellow circle*). A magnification of the structures of interest is provided for each slice

An important tool for visualization of the trigeminal nerve and detection of pathologies in the oral and maxillofacial regions is 3 Tesla (3T) MRI. Even without the use of contrast agent, 3T MRI is clear enough for highly defined and detailed visualization of the IAN and IANC. The best sequences for this are the non contrast-enhanced fat-saturated T1- and T2-weighted (T1w/T2w) images [2]. When evaluating perineural spread of malignancies, 3T MRI is performed with contrast agent [6]. Alternatively, the course of the IAN and the mandibular trigeminal division from its exit from the foramen ovale can be followed with high-resolution T1w sequences and post-contrast fat-suppressed T1w images (Fig. 3) [6, 21]. In this respect, Tabuchi et al described that the enhancement of the normal trigeminal nerve seen on gadolinium-enhanced MRI (Gd-MRI) is caused by the vascular permeability of the blood vessels of these nerves [40].

Deng et al performed high-resolution MRI using a T1-weighted 3-dimensional magnetisation-prepared rapid gradient-echo (3D MP-RAGE) sequence before and after the administration of a contrast agent (gadolinium-DPTA). This makes visualization of normal and pathological nerve structures possible (e.g., mandible fractures, cystic lesions) [11]. Recently, conventional mandibular MRI was experimentally modified to allow for diffusion tensor tractography (DTT) and phase-contrast magnetic resonance angiography (PC-MRA) [23, 36]. DTT can visualize the individual nerve fibers and their direction and integrity in healthy volunteers [6, 23]. Sakamoto et al performed the first study to apply PC-MRA to the mandible to visualize the inferior alveolar artery (IAA) as a line of high intensity [36]. Another modification of the conventional MRI is dental MRI. Nasel et al designed a high-resolution gradient-echo sequence, with a spectral fat suppression pre-impulse, using an 1T MRI scanner with a standard neck-quad coil. Since scanning is done in the axial plane, metallic restorations in the teeth do not interfere because the anatomical structures of interest lie below the occlusal plane [26]. Dental MRI can reliably visualize the whole neurovascular bundle, but neural and vascular structures within the IANC cannot be identified separately. Intravenous MR contrast agents could be helpful here [26]. In the future, it might become possible to visualize the IAN using manganese-enhanced magnetic resonance imaging (MEMRI). This technique could already successfully visualize the optic nerve in rodents, but further research is needed for IAN visualization and application in humans [4, 18, 38, 42].

**Fig. 4:**
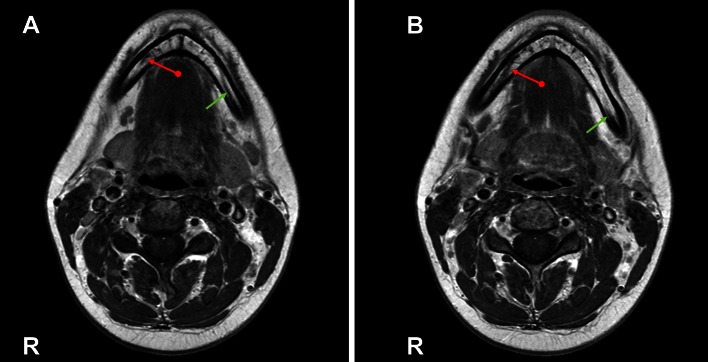
Axial 3T-MRI images (T1w turbo spin-echo sequence with contrast agent) showing neuritis of the right IAN at molar and premolar levels (*red arrow* with *round end*, **b**). The neuritis can be identified by the asymmetrical thicker appearance of the IAN at the *right side* (**b**), compared to the normal left IAN (**a**) (*green arrow*, **a**)

An important clinical application of MRI is the integration of the IAN anatomy in the treatment planning of orthognathic surgery, traumatology, cysts, tumors, M3 removal, and implantology [9, 11]. In this way, the planning is refined and the risk of perioperative IAN injury can be reduced [11]. Also in cases where the IANC cannot be identified on CBCT images, MRI is an alternative imaging modality [9]. Furthermore, MRI can diagnose pathologies of the IAN such as neuritis (Fig. 4). DTT can track the IAN fibers in healthy mandibles [23, 40]. PC-MRA can be used to diagnose and plan treatment in lesions adjacent to the IAA, for inflammation of the mandible, mandibular tumors, and invasion by oral cancers [36]. Dental MRI could become an alternative to panoramic radiography or CBCT due to the adequate visualization of the whole neurovascular bundle, the short duration, and the sufficient resolution for planning oral surgery [26]. A novel application of 3T MRI is to determine the prognosis of IAN sensory disorders after M3 removal, by evaluating the relative signal intensity (RSI) of the IAN in the early post-operative period. Cassetta et al. concluded that there is a statistically significant difference in RSI among patients with a different grade of neurosensory impairment after M3 removal [7]. Finally, MRI is also used to evaluate regions of the TMJ, salivary glands, the floor of the mouth, and the paranasal sinuses [2].

The main advantage of MRI is that it can visualize the whole course of the IAN directly because of the excellent soft-tissue contrast [9, 11, 26]. In this way, it provides information on the relationship and distances between the IAN and the neighboring anatomical structures [11, 26]. An additional benefit when using contrast agent is the possibility to perform a subtraction operation using the data with and without contrast agent. This may help show pathological changes [11]. A final advantage of MRI is the easier detection of the IAN, the mental foramen, and the mandibular foramen in MRI images compared to CBCT, also for non-radiologists (e.g., dentists) [9].

A disadvantage of high-resolution MRI is the long imaging time that can cause movement artifacts. This is also a problem in older patients with tremor [2, 11, 36]. However, in dental MRI the scanning time is short (about 5 min 40 s) [26]. Finally, intra-oral metallic structures (dental fillings, dental implants) limit the image quality with ferromagnetic materials (e.g., amalgam, gold, cobalt-chromium) causing distortion of the magnetic field and deformation of the images. Non-ferromagnetic materials do not create such detrimental artifacts (e.g., titanium) [2, 24].

## CBCT/CT

The main difference between CT and CBCT is the conical shape of the X-ray beam in CBCT, compared to the 2-dimensional fan beam of X-rays used in conventional CT [14, 17, 31]. This explains the isotropic nature of acquisition and reconstruction in CBCT systems. Because each voxel is isotropic, the spatial resolution remains identical in all planes. In addition, CBCT voxels are very small compared to CT voxels, resulting in a very high spatial resolution of CBCT, higher than that of conventional CT [14].

Kim et al developed a novel method to automatically extract the IANC in CT images [17]. In experimental results using 10 clinical datasets, this method could identify the IANC accurately. In contrast to most previous researches on the IANC detection, this method is completely automatic and does not require any intervention from the user such as a manual trace or the detection of the initial points of the IANC [35]. Also, this method has the highest segmentation accuracy [17].

**Fig. 5:**
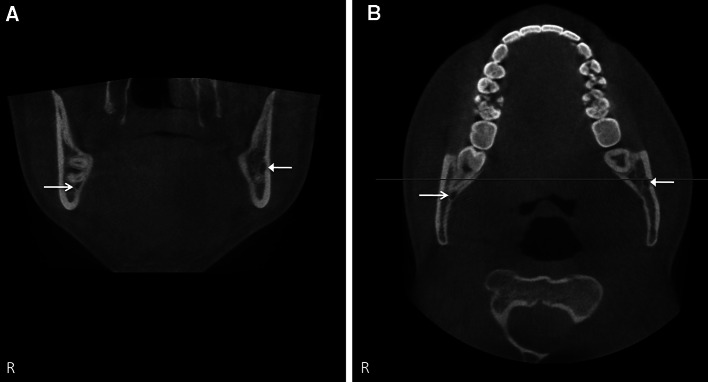
Coronal (**a**) and sagittal CBCT images (**b**) of impacted M3 right in close relation to the IANC (*arrow* on *right side*). The IANC on the *left side* is situated close to the buccal cortex (*arrow* on *left* *side*)

**Fig. 6:**
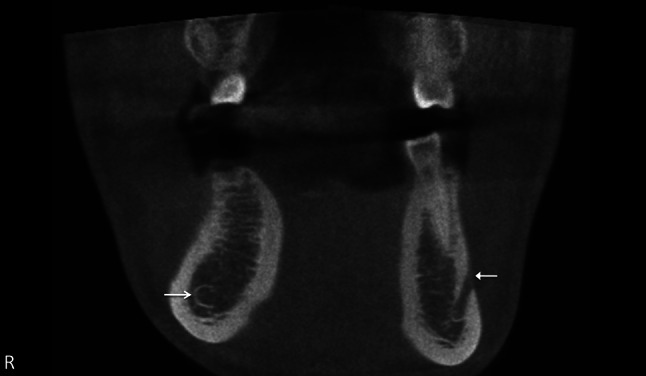
Coronal CBCT slice at level of premolars, taken for pre-implant planning on the *right side*, showing the IANC (*right side*, *arrow*) and mental foramen (*left side*, *arrow*)

Among the clinical applications of CBCT is the preoperative evaluation and the risk assessment of M3s and dental implants (Figs. 5, 6) [21, 32, 43]. CBCT is suggested to be the main method for radiographic pre-implant planning [16, 43]. It is also used to evaluate the relationship between the IANC and impacted M3s, mostly in patients in whom the IANC and M3 show a close relation on the initial panoramic radiography (e.g., superimposition of IANC and M3, increased radiolucency, interruption of the radiopaque border of the IANC, diversion or narrowing of the IANC) [22, 43]. Other applications of CBCT are the 3-dimensional imaging and treatment planning in orthognathic surgery, traumatology, and cleft patients. CBCT is also a useful tool in oral and maxillofacial diagnosis and for the evaluation of the bony component in temporomandibular joint disorders [32]. Furthermore, CT is required in critically ill patients and trauma patients who may not tolerate an MR study [6]. CT and MRI are complementary in the evaluation of the peripheral segments of the trigeminal nerve. CT is appropriate for visualization of the skull base neurovascular foramina and for diagnosing primary bone and fibro-osseous conditions, while MRI is preferred to assess the soft tissue components [5, 6]. Clinical implications for the automatic extraction method are implant placement and orthognathic surgery [17, 32].

The most important advantages of CBCT are the low radiation exposure (patients are exposed to approximately 20 % of the radiation of a conventional CT, similar to the exposure from a full-mouth periapical series) and the high accuracy and spacial resolution [21, 23, 32]. It is possible to reconstruct 3-dimensional images and obtain information on the buccolingual bone width [16, 17, 43]. Both scanning time in CBCT and the processing time in the automatic extraction method are short [16, 17].

The main disadvantage of CBCT is that it cannot directly visualize the IAN itself, since it cannot obtain accurate soft tissue information [9, 14, 17, 21]. As a result, it is not always possible to conclude that the small branches of the IANC seen on CBCT also contain branches from the IAN [43]. Even CT images in a soft tissue window cannot clearly depict the IAN because of the very poor detail resolution [21]. Another problem is dental artifacts which can severely limit the image quality [16, 21]. The disadvantage of the automatic extraction method is that it might fail sometimes. This could be when a considerable part of the IANC is missing in the CT images (often due to osteoporosis), when the foramens are not scanned due to a small field of view (FOV) of the CT scanner used or after mandibular surgery, e.g., BSSO [17, 30].

## Panoramic radiography

Only structures located within the horseshoe-shaped tomographic plane of panoramic radiography are well delineated and those in front or behind of that plane are blurred [39]. Since panoramic radiography is a 2-dimensional examination, it can provide valuable information about the relationship to the IANC in the vertical plane, but not in the horizontal plane [16]. Often these radiographs are sufficient for dental imaging [39].

Clinically, panoramic radiography is mostly applied to evaluate the position of the IANC and to assess the risk of IAN injury during M3 surgery, based on the presence of radiographic predictors [21, 22]. Periapical radiographs are not advised for preoperative risk assessment of IAN injury, since they cannot always provide reliable and reproducible information [21].

Advantages of panoramic radiography are the wide coverage of oral structures, the low radiation dose, and the relatively inexpensive equipment [16, 39].

Besides the lack of 3-dimensional information and the inability to visualize the IAN itself, the lower resolution, the higher distortion, and the possibility of phantom images are major drawbacks of this technique [15, 16, 39].

## Ultrasonography

Chanpong et al developed a technique for ultrasonographic visualization of the IAN using an 8- to 15-MHz hockey stick-shaped transducer, attached to an ultrasound machine. The probe is placed axially along the occlusal mandibular surface against the pterygomandibular raphe and is rotated transversely until the ramus can be identified. Then the probe is moved upwards until the IAN can be visualized by its fascicular appearance. In this way, the IAN could be followed until it enters the foramen ovale. The IAA was only visible in 6 of the 40 cases. A possible explanation for this might be compression of the artery by the transducer [8].

The main clinical application for ultrasound is the ultrasound-guided IAN block. This may improve success rates for IAN injection [8]. Ultrasonography has not been investigated as a potential preoperative risk assessment tool for the IAN [21]. It might be applied for intraoral measurements of the IANC, as a diagnostic tool before implant placement [8, 20].

The main advantage of ultrasonography is that it can visualize the IAN. As a result, the number of IAN block failures, intraneural injections, and vascular puncture is reduced when using ultrasound guidance. Additionally, it is comfortable for the patient (similar to a bite block) [8, 21]. There is no evidence yet about a faster onset of the IAN block or a possible dose reduction when using ultrasound guidance [8].

The main disadvantage of ultrasonography is that it is impossible to visualize the IAN inside the canal as bone and teeth affect the echogenic signal. It can only image the IAN in the ramus region. Consequently, this technique cannot be used in the preoperative planning and risk assessment of M3 removal and BSSO [8, 21].

## Endoscopy

In oral and maxillofacial surgery, endoscopy is done using a rigid endoscope with support and irrigation sheaths. Support endoscopy is a tool for macroscopic observation of a superficial site at a certain distance. It is used for visualization in minimally invasive surgery. Immersion endoscopy provides microscopic observation under continuous irrigation, mostly in contact with surface structures with difficult access. This is a diagnostic tool, since it can be used for detailed observation under high magnification. The combination of both tools enables the surgeon to get a complete overview and detailed information of a certain area [3].

Endoscopic inspection can clarify the course and microstructure of the neurovascular bundle, the quality of osseous tissue, the vascularization, and the existence of alveolar wall defects [13]. It enables visualization of the IAN during and after M3 removal by changing the view angle of the surgical site. In case of an exposed neurovascular bundle, endoscopy also serves as a documentation tool after tooth removal. In addition, lingual nerve exposure can be detected. This is seen when the lingual periosteum is exposed (e.g., after fracture of the lingual wall due to M3 removal). Endoscopy can also be used for the assessment of implant sites and the possibility for immediate implant placement [3]. Besides these diagnostic purposes, endoscopic techniques also have therapeutic goals. They are successfully applied during elevation of the maxillary sinus and are a useful aid in surgically assisted rapid palatal expansion, arthroscopy of the temporomandibular joint, paranasal sinus surgery, and condylar fracture repair [12, 13, 25, 34].

The main advantage of endoscopy is that it enables direct visualization of the IAN and other anatomic structures of interest. It can improve the quality and reduce the invasiveness of oral and maxillofacial surgery since the required incisions are smaller and less degloving is needed [3, 12].

A disadvantage is the need of specialized and more expensive equipment. Also therapeutic interventions using endoscopic guidance have a longer duration and a prolonged learning curve [34].

## Conclusion

High-resolution MRI is the most commonly used method for direct visualization of the IAN [9, 11, 23]. Recently, there have been some (experimental) modifications to the conventional MRI in the form of Diffusion Tensor Tractography (DTT), Phase-Contrast Magnetic Resonance Angiography (PC-MRA), and dental MRI [23, 26, 36]. DTT provides image contrast by diffusion anisotropy and can visualize the individual nerve fibers and their pattern of running [23]. PC-MRA can depict the inferior alveolar artery [36]. Dental MRI can quickly and reliably visualize the whole neurovascular bundle [26]. In the future, this could gain importance in the preoperative risk assessment. Furthermore, an intraoperative application of MRI to visualize the IAN during surgery would be useful, but more research in this field is needed.

Although CBCT is an ideal imaging modality in the daily oral and maxillofacial practice, it can only show the bony wall of the IANC and cannot visualize the nerve itself. Nevertheless, CBCT is the main method for radiographic pre-implant planning nowadays and it is an important tool to evaluate the relationship between (impacted) M3s and the IAN before M3 removal [16]. The automatic extraction of the IANC using CT can delineate the path of the IANC. This is helpful as a surgical planning tool [17].

Panoramic radiography is useful in daily practice as it provides an overview of the important oral structures and is widely used [21]. It can be used to evaluate the position of the IANC and to assess the risk of IAN injury during M3 surgery, based on the presence of radiographic predictors [21, 22]. However, panoramic radiography lacks 3-dimensional information and cannot visualize the nerve itself [16, 23].

Ultrasound and endoscopy are the methods that can directly visualize the IAN [3, 8]. For now, they are mostly applied on a smaller scale or even in an experimental setting, but in the future they could gain importance. Ultrasound cannot be used for the preoperative risk assessment since the bone and teeth interfere with the echogenic signal, but it serves as a guidance for IAN injection [8, 21]. Endoscopy can visualize the IAN during and after M3 removal [3, 8].
